# Promotion of Pre-natal Education Courses Is Associated With Reducing the Rates of Caesarean Section: A Case-Control Study

**DOI:** 10.3389/fpubh.2021.666337

**Published:** 2021-05-28

**Authors:** Yunhui Tang, Jing Gao, Liping Sun, Yifei Gao, Fang Guo, Qi Chen

**Affiliations:** ^1^The Hospital of Obstetrics & Gynaecology of Fudan University, Shanghai, China; ^2^Department of Obstetrics & Gynaecology, The University of Auckland, Auckland, New Zealand

**Keywords:** caesarean section rate, pre-natal education course, delivery mode, retrospective study, attendance

## Abstract

**Objective:** The number of women having a caesarean section has significantly increased worldwide, in particular in China. Maternal requestion makes a moderate contribution to this increased rate in China. Reducing the caesarean section rate is now becoming a big challenge to midwives and obstetricians as well as health policymakers in China. Our recent survey found that pre-natal education course had some positive effects on the reduction of caesarean section on maternal request. However, pre-natal education course is relatively new in China. In this study, we investigated whether pre-natal education course influences delivery mode in the largest tertiary women's hospital in China.

**Methods:** In this retrospective study, during the study period, 644 pregnant women attended a pre-natal education course and 4,134 pregnant women did not. Data on maternal age, parity, gravida, delivery mode, delivery weeks, birthweight, gestational age at attending pre-natal education course and maternal body mass index before pregnancy were collected and analysed.

**Results:** The numbers of women who attempted vaginal delivery were significantly higher in women who attended a pre-natal education course, compared to women who did not (87 vs. 60%). In addition, the rate of caesarean section on maternal request was 23% in women who attended a pre-natal education course.

**Conclusion:** Attendance of a pre-natal education course influences the mode of delivery and reduces the unnecessary caesarean section in China. Our findings suggest that the promotion of pre-natal education courses is important to reduce the higher caesarean section rate in China, by midwives or obstetricians or health policy-makers as part of China's strategy.

## Introduction

Increasing evidence shows that the global caesarean section rate has significantly increased (1), although the World Health Organisation (WHO) recommends that there is a necessity for having a medical indication of caesarean section (WHO Statement on Caesarean Section Rate, 2015). Based on the WHO report, China is one of the countries with a higher caesarean section rate (around 35–50%, regionally dependent), in comparison to other countries (1–3). The predominant reason for this trend may be that women have distress and anxiety of childbirth as well as psychological stress during labour and birth (4, 5). This results in an increase in the number of caesarean section on maternal request in the last decade in China (2, 3). Our recent survey on investigating factors associated with caesarean section on maternal request reported 46% of pregnant women to request caesarean section (6), which may be higher than other ethnic groups. There are 5–40% of pregnant women in western countries having childbirth fear (7, 8) and Chinese women have moderate levels of childbirth fear and anxiety (9). This fear and anxiety were increased by pregnant women with lower educational levels and younger maternal age, as well as no support from the husband during labour and birth (9). In addition, due to the limitation of maternal care facilities, failure of hospitals to support normal deliveries including a supportive birthing environment can result in maternal stress and anxiety during labour and birth (10). A satisfying birth environment can minimise maternal stress and anxiety during labour and birth and support physiologic birth (10). Finally, due to the One-Child Policy in China, many pregnant women and their families prioritise baby's safety and this consequently increases the rate of caesarean section on maternal request (11).

Many factors, such as maternal facilities, the relationship of patient-doctor and pre-natal education course are associated with the higher caesarean section rate and it may vary by ethnicities and regions. In most developed countries, pre-natal education courses have become a part of antenatal care for a long time. This is because that such a course can provide much information about labour and birth such as the process of labour and birth process and how to manage pain relive, how to breastfeeding after birth, and care of the new baby (12). Attendance of a pre-natal education course reduces the fear of labour and increase childbirth-related maternal self-efficacy (13). The attendance rate was 33% in Canada (14) and 84% in nulliparous women in Australia (15). However, such advanced courses are not performed in most maternity facilities in China yet. Furthermore, the attendance of pre-natal education courses is <20% in hospitals where they run pre-natal education courses. The significantly lower attendance is mainly because such pre-natal education courses are new to most pregnant women and their families, and it has not been strongly recommended yet, by social media, midwives or obstetricians. In addition, studies about the effect of pre-natal education course on pregnant women's expectation of birth are limited (16). Our recent survey found that attendance of a pre-natal education course had some positive effects on the reduction of caesarean section on maternal request (6). Therefore, in this retrospective study, we investigated whether pre-natal education course influences the mode of delivery in one of the largest tertiary women's hospitals in Shanghai, China.

## Methods

This retrospective study was approved by the ethics board of The Hospital of Obstetrics & Gynaecology of Fudan University (Reference No. 2017-04).

### Patient and Public Involvement

No patient and public involvement in this study.

### Study Design and Participants

During the study period from June 2018 to May 2019, there were 4,778 deliveries in the Huangpu campus of The Hospital of Obstetrics & Gynaecology of Fudan University. Of these pregnancies, 644 pregnant women went to a pre-natal educational course.

The pre-natal education course described in our study started in 2013 in our hospital. It is a free half-day intensive one by one course for pregnant women between 36 and 37 weeks of gestation in our hospital. The course is led and run by experienced and specialised midwives in our hospital. It is common that either husbands or parents (in particular pregnant women's mothers) come along with the pregnant women. Course information focuses on supporting pregnant women to prepare for labour and normal births including the role of the father during labour and birth, labour coping skills and management of pain during labour and birth.

The Hospital of Obstetrics & Gynaecology of Fudan University is located in Shanghai, the wealthiest city in China, and is a leading provider of tertiary maternity care with two campuses in China. Our hospital has more than 12,000 deliveries in a year in total.

Data on maternal age, parity, gravida, delivery mode, delivery weeks, birthweight, gestational age at attending to pre-natal education course and maternal body mass index (BMI) before pregnancy were collected and analysed. The indications of the caesarean section including an emergency caesarean section were also collected in pregnant women who attended a pre-natal education course.

### Statistical Analysis

The statistical difference in planned vaginal delivery or caesarean section between pregnant women who attended a pre-natal education course was assessed with the Chi-square test (or Fisher's exact test) using the Prism software package. *P*-values of <0.05 were considered significant.

## Results

During the study period of 1 year, 644 pregnant women attended a pre-natal education course in our hospital. The attendance rate was only 13.5% (644 out of 4,778 deliveries in total). Clinical characteristics of pregnant women who attended a pre-natal education course are summarised in Table 1. The mean maternal age was 31 years and 14% of pregnant women had previous experience of live birth.

**Table 1:** Clinical characteristics of pregnant women who attended to a pre-natal education course.

**Maternal age (years, mean/SD)**	**31.02 ± 3.72**
Parity (*n*, %)	
*P* = 0	552 (85.7%)
*p* ≥ 1	92 (14.3%)
Gravida (*n*, %)	
*G* = 1	524 (81.4%)
*G* ≥ 2	120 (18.6%)
Delivery week (mean/SD)	39^+^5 (± 2^+4^)
Birthweight (g, mean/SD)	3,446 ± 1,566
BMI (Kg/m^2^, mean/SD)	21.22 ± 2.91

Of 644 pregnant women who attended a pre-natal education course, there were 559 (86.8%) pregnant women who attempted to have a vaginal delivery (referred to as planned vaginal delivery). While there were 85 (13.2%) pregnant women with planned caesarean section (Table 2). In contrast, during the study period, 4,134 pregnant women did not attend a pre-natal education course. Of them, 2,470 (59.7%) pregnant women attempted to have a vaginal delivery (referred to as planned vaginal delivery), which was significantly lower in comparison with pregnant women who attended a pre-natal education course (Table 2, *p* < 0.0001). The planned caesarean section (including an emergency caesarean section) in pregnant women who did not attend a pre-natal education course was 40.3%, which was significantly higher than pregnant women who did (Table 2, *p* < 0.0001).

**Table 2:** The comparison of delivery mode between women who attended pre-natal education course and who did not.

	**Women who attended to a pre-natal education course (*n* = 644)**	**Women who did not attend to a pre-natal education course (*n* = 4,134)**	***P*-value**
Planned vaginal delivery (*n*, column %)	559 (86.8%)	2,470 (59.7%)	*P* < 0.0001
Planned caesarean section (*n*, column %)^*^	85 (13.2%)	1,664 (40.3%)	

* *Including emergency caesarean section*.

We then analysed the indications for a planned caesarean section in pregnant women who attended a pre-natal education course (Table 3). Overall, caesarean section on maternal request was the single most frequent reason for caesarean section (23.5%), followed by previous caesarean section (15.2%), complications of pregnancy (14.1%) and macrosomia (12.9%).

**Table 3:** Indications for planned caesarean section in pregnant women who attended to a pre-natal education course.

**Indications**	**Total *n* = 85**
Caesarean section on maternal request	20 (23.5%)
Previous caesarean section	13 (15.2%)
Complications of pregnancy	12 (14.1%)
Macrosomia	11 (12.9%)
Oligohydramnios	9 (10.5%)
*In vitro* fertile (IVF)	7 (8.2%)
Foetal distress	4 (4.7%)
Chorioamnionitis	3 (3.5%)
Others	6 (7%)

We next analysed the rate of emergency caesarean section in pregnant women who attempted to have a vaginal delivery. In pregnant women with a planned vaginal delivery who attended a pre-natal education course (*n* = 599), 97 (17%) pregnant women ended up with an emergency caesarean section. In contrast, 286 (11.6%) pregnant women with a planned vaginal delivery who did not attend to a pre-natal education course ended up with an emergency caesarean section, which was significantly lower (Table 4, *p* = 0.0003). We then analysed the indications for emergency caesarean section in pregnant women who attended a pre-natal education course (Table 5). Overall, foetal distress was the single most frequent reason for emergency caesarean section (*n* = 39, 40%), followed by chorioamnionitis (*n* = 27, 28%) and maternal complications of pregnancy (15%).

**Table 4:** The comparison of emergency caesarean section in planned vaginal delivery between women who attended to a prenatal education course and who did not.

**Planned vaginal delivery**	**Women who attended to a prenatal education course (*n* = 599)**	**Women who did not attend to a prenatal education course (*n* = 2,470)**	***P*-value**
Ended up with vaginal delivery (*n*, column %)	462 (83%)	2,184 (88.4%)	*P* = 0.0003
Ended up with caesarean section (*n*, column %)	97 (17%)	286 (11.6%)	

**Table 5:** Indications for emergency caesarean section in women who attended to a pre-natal education course.

**Indications**	**Total *N* = 97**
Foetal distress	39 (40%)
Chorioamnionitis	27 (28%)
Maternal complications	15 (15%)
Failure of delivery	9 (9%)
Oligohydramnios	7 (7%)

## Discussion

### Main Findings

In this retrospective study, we found that the attendance of a pre-natal education course is very low in a leading provider of tertiary maternity care in Shanghai, China, in comparison to western countries reported in the literature. However, the attendance of a pre-natal education course can significantly influence the mode of delivery. The rate of attempting vaginal delivery was significantly higher in pregnant women who attended a pre-natal education course, compared to pregnant women who did not. In addition, attending a pre-natal education course significantly reduced caesarean section on maternal request.

### Strengths and Limitations

China has a higher caesarean section rate worldwide. Whether attendance of a pre-natal education course could reduce the caesarean section rate has not been fully investigated, as pre-natal education course is relatively new in China. Our hospital provides a one on one pre-natal education course since 2013.

The limitations of this study include (1) this retrospective study was performed in a single tertiary women hospital. Sample size and regional differences may result in a bias. (2) The aims of our pre-natal education course were not associated with an obstetric intervention. Therefore, it should be aware that we did not state the association of pre-natal education course and perinatal outcomes in this study.

### Interpretation

It is well-reported that China has a higher caesarean section rate around 35% to 50%, dependent on the regions, in comparison to other countries (2, 3). In addition, studies including our recent survey reported higher rates of caesarean section on maternal request (38–46%) in China (2, 6). Reducing the caesarean section rate is now becoming a big challenge to midwives and obstetricians as well as health policymakers in China. Interestingly, our recent survey found that the attendance of a pre-natal education course could reduce the rate of caesarean section on maternal request (6).

Pre-natal education courses are well-designed programmes that aim to reduce perinatal morbidity and mortality in mothers and children for many years in middle and high-income countries (17). Pre-natal education courses also support pregnant women to prepare for labour and birth and include information on the labour and birth process, pain management, breastfeeding, and care of the newborn (12, 18, 19). A recent randomised trial study reported that pre-natal education course can increase childbirth self-efficacy (20) and can impact maternal birth expectations (18). Therefore, pre-natal education courses can support pregnant women to lessen their anxiety about birthing and to build confidence to give birth. In this retrospective study, we found the number of pregnant women attempting to have a vaginal delivery who attended a pre-natal education course was significantly higher, compared to pregnant women who did not attend a pre-natal education course (86.8 vs. 59.7%). Our finding is also supported by other studies suggesting pre-natal education courses have benefits on increase in vaginal delivery (6, 14). Taken together, our result suggests that attendance of a pre-natal education course can influence the mode of delivery.

It has been reported that an increased rate of caesarean section on maternal request contributes to the overall increased caesarean section rate. Worldwide, the estimated rate of caesarean section on maternal request was 10-20% in Northern Europe, the United States, Sweden and Australia (21–24). Recent studies reported that the caesarean section on maternal request is high in China, around 38–46% (regionally dependent) (2, 6). However, in our current study, we found a lower rate of caesarean section on maternal request (23.5%) in pregnant women who attended a pre-natal education course, even though the caesarean section on maternal request was a leading reason for caesarean section. This rate is significantly lower than other studies performed in China (2, 6) and is similar to studies performed in western countries (21–24). Collectively, our finding further suggests that the attendance of a pre-natal education course can significantly reduce the rate of caesarean section on maternal request.

In order to reduce maternal and foetal morbidity and mortality, an emergency (unscheduled) caesarean section is recommended. Recent studies reported that 15–23% of pregnant women who attempted to have a vaginal delivery ended up with an emergency caesarean section in Australia, United Kingdom and Palestine (25–27). Consistent with these studies, our current study found that 17% of pregnant women who attended a pre-natal education course ended up with an emergency caesarean section. Foetal distress was the single most frequent reason for emergency caesarean section, followed by chorioamnionitis and complicated pregnancy (such as preeclampsia and gestational diabetes mellitus). Although we found that this rate was slightly higher than that in pregnant women who did not attend a pre-natal education course (11.6%), this could be because most pregnant women who did not attend a pre-natal education course selected a planned caesarean section if they have some potential risks for vaginal delivery.

Studies have shown a strong bias towards more well-educated, wealthier women attending pre-natal education sessions in the literature. In this study we cannot analyse what factors could affect the attendance of a pre-natal education course due to the availability of data, however, our recent survey clearly reported that parity, gravida, maternal age and educational levels were not associated with attendance of a pre-natal education course (6). The lower attendance of a pre-natal education course in our hospital is because of the short duration of this course, around 5 years and it has not been strongly recommended yet, by a midwife or obstetrician. To date, there are still not many level 3 maternal care facilities providing a pre-natal education course in Shanghai. Although our pre-natal education course was not designed to reduce the rates of obstetric intervention, with the goal of reducing unnecessary caesarean section rate at the population level, we hope that our findings will encourage obstetricians and midwives, as well as health policy-makers to recommend pre-natal education courses to pregnant women.

There are some limitations in our current study. First, our study was done in one single leading provider of tertiary maternity care and was not a randomised clinical trial (RCT) to draw the conclusion. Further research in a multicentre in China is needed to confirm our findings. Secondly, data on indications of planned caesarean section in pregnant women who did not attend a pre-natal education course were not available. We were not able to compare the difference in indications of the planned caesarean section between the two groups.

## Conclusion

In this study of pregnant women who attended a pre-natal education course at a tertiary maternal hospital in Shanghai, China, we found that the numbers of pregnant women with vaginal delivery were significantly increased, compared to pregnant women who did not attend a pre-natal education course. In addition, attendance of a pre-natal education course significantly reduced the rate of caesarean section on maternal request. Our findings suggest that in order to reduce the overall caesarean section rate in China, pre-natal education courses should be actively promoted by midwives or obstetricians or health policy-makers, as part of China's strategy.

## Data Availability Statement

The raw data supporting the conclusions of this article will be made available by the authors, without undue reservation.

## Ethics Statement

The studies involving human participants were reviewed and approved by the ethics board of The Hospital of Obstetrics & Gynaecology of Fudan University. Written informed consent for participation was not required for this study in accordance with the national legislation and the institutional requirements.

## Author Contributions

All authors were involved in the drafting, editing, and approval of the manuscript for publication. In addition to this, each author contributed to the following work. YT, LS, and YG: collected the data reported in this work. JG: assistance with data analysis. FG and QC: designed the study and wrote the manuscript draft. QC: completed the final manuscript.

## Conflict of Interest

The authors declare that the research was conducted in the absence of any commercial or financial relationships that could be construed as a potential conflict of interest.
